# What a pickle—a metagenomic perspective on the cucumber fermentation

**DOI:** 10.3389/fmicb.2026.1809866

**Published:** 2026-04-28

**Authors:** Aleksandra Glapa-Nowak, Jan Krzysztof Nowak, Szymon Kurek, Jarosław Walkowiak

**Affiliations:** Department of Pediatric Gastroenterology and Metabolic Diseases, Poznan University of Medical Sciences, Poznań, Poland

**Keywords:** brine, microbial diversity, microbiota, nanopore, organic

## Abstract

Food fermentation involves an interplay between multiple strains and species. This delicate composition during fermentation has been investigated so far using both classical and molecular methods; however, the results remain difficult to interpret. In this perspective article, we discuss the spontaneous fermentation of cucumber from organic and commercial cultivation (from 1st day to 90th day) based on our preliminary data from a nanopore sequencing study. The present study is the first to report the occurrence of coagulase-negative cocci in cucumber fermentation [*Staphylococcus saprophyticus* (0.01%) and *Staphylococcus schleiferi* (0.03%)]. Furthermore, we conclude that own-cultivation cucumbers may exhibit a lower incidence and diversity of phages, which have practical implications for designing future studies as well as for direct consumers. Our data also show that, even in the absence of phages (own-cultivation cucumbers <1%), lactic acid bacteria dominance occurs, which contrasts with previous conclusions and contributes to the discussion on the role of phages in maintaining the balance between *Enterobacteriaceae* and lactic acid bacteria in plant fermentation. The powerful metagenomic approach provides a broader understanding of the day-to-day and sample-to-sample diversity within microbiome communities. The maturity of the fermentation product may play a significant role in exerting specific biological actions. This should be accounted for before planning an intervention study.

## 1 Introduction

Cucumber (*Cucumis sativus* L.) belongs to the top vegetables in the fermentation industry. The intake of fermented foods varies by geographical region, ranging from 5% of daily intake in one region up to 40% in another (El Sheikha and Hu, 2020; Tamang, 2009). In Poland, cucumbers are the most popular fermented vegetables, and almost 70% of households admit to consuming them either weekly or several times a week (Wiśniewska et al., 2022). The majority (63%) declared that they prepared homemade products, and over 40% of these came from vegetables grown on their own plantations (Wiśniewska et al., 2022).

The microbial composition after the spontaneous fermentation of cucumbers depends on the microorganisms present on the surfaces of raw vegetables, which can vary with harvest season, origin, size, and farming practices (Reina et al., 2005; Leff and Fierer, 2013). Washing vegetables before fermentation reduces the microbial count and promotes the growth of lactic flora (Swain et al., 2014). Additional factors, such as salinity, temperature, and access to oxygen, also play a crucial role in fermentation (Mcfeeters, 2004; Stoll et al., 2020). Garlic used in pickled products can inhibit spoilage due to its sulfur compounds (Chakraborty and Roy, 2018). Similarly, dill or mustard seeds inhibit yeast growth and promote the growth of lactic acid bacteria (LAB), the key players in fermentation (Reina et al., 2005; Swain et al., 2014; Steinkraus, 2002). Of these, the most frequent genera are *Lactobacillus*, *Lactococcus*, *Leuconostoc*, *Pediococcus*, *Streptococcus*, *Enterococcus*, and *Weissella* (Wang et al., 2021). Most of those (*Lactobacillus*, *Leuconostoc*, *Pediococcus*, *Streptococcus*, *Enterococcus*) are considered probiotics in the intestine (Fijan, 2014).

Researchers have known for over a century that viruses, along with bacteria, are present in food fermentations, such as dairy products (Marcó et al., 2012; Pujato et al., 2019). Nevertheless, scientists identified bacteriophages in cucumber fermentations only recently (Lu et al., 2020). The dominant viral order in fermented products is *Caudovirales*, of which the most prevalent (~70%) are *Myoviridae*, *Podoviridae*, and *Siphoviridae* (Park et al., 2011; Ledormand et al., 2021). Studies have shown that phages regulate the abundance and distribution of bacteria and affect the fermentation process (Lu et al., 2020). Previously, research on viral communities was limited due to a lack of methodology, as viruses require host cells to be cultivated, and not all host microorganisms can be cultured in standard laboratory conditions (Park et al., 2011). Furthermore, viruses lack universal markers such as the 16S rRNA gene used for bacterial identification (Sausset et al., 2020). Recent advances in technology have facilitated direct whole metagenome sequencing, which has expanded our understanding of viral diversity (Park et al., 2011). Although the available phage genomes database is rapidly growing, doubling the number of complete phage genomes in 5 years, their exact function in food fermentations remains largely unknown (Ledormand et al., 2021).

It has been suggested that the abundance of phages against *Enterobacteriaceae* and many other Gram-negative bacteria may be the primary driver in eradicating these bacteria and promoting the dominance of LAB (Lu et al., 2020). To verify this hypothesis, we employed the nanopore technique to investigate the metagenetic changes during non-commercial-scale cucumber fermentation, as per the traditional Polish recipe, in low-salt brine. In the procedure, we involved cucumbers from our own-cultivation as well as commercially available plants, sampled at several time points until 90 days.

## 2 Materials and methods

### 2.1 Samples preparation

One sample with own-cultivated cucumber (growing season 2021) was fermented for 1 year and included in the study. Fresh cucumbers (*Cucumis sativus* L.) from own organic cultivation (growing season 2022) were washed and placed in seven 330 mL glass jars. Commercial cucumbers from local grocery store (growing season 2022) were washed and placed in four glass jars. Every jar contained approximately 190 g of cucumbers (all ~8 cm), garlic, dill, horseradish and mustard seeds, according to a local recipe. Around 150 mL of brine (containing 1.5% NaCl) was added to each jar (1 cm from the brim). The jars were covered and left to ferment at room temperature (about 22–27 °C). The brine samples were collected by sterile syringe and immediately stored at −20 °C in sterile tubes (Vacuette^®^ tube 9 mL Z No Additive). The sampling was done after vigorous inverting of the jar at least 10 times. The sampling schedule for own-cultivation cucumber fermentation was as follows: 1st day, 3rd day, 4th day, 10th day, 2nd week, 1st month, 3rd month of fermentation. The sampling schedule for commercial cucumber fermentation was as follows: 2nd day, 3rd day, 4th day and 7th day. Each jar was sampled once at each assessment point.

DNA was isolated from 3 mL of brine using Bead-Beat Micro AX Gravity (A&A Biotechnology, Gdansk, Poland). The homogenization step was carried out using zirconium beads and Digital Disruptor Genie (Scientific Industries, New York, United States) at maximum frequency, over 1 min.

To increase concentration and purity of the samples prior to sequencing, the samples were further processed with Clean-up AX kit (A&A Biotechnology, Gdansk, Poland). DNA concentration and purity were assessed using Qubit 4 and Nanodrop (Thermo Fisher).

### 2.2 Sequencing

Sequencing was achieved on MinION Mk1B (Oxford Nanopore Technologies, Oxford, United Kingdom) in one batch, using a R9.4.1 FLO-MIN106 flowcell, primed with FLP002 kit. The rapid barcoding sequencing kit (SQK-RBK004) was used for library preparation. MinKNOW 4.3.12 qscore was set at 7 and high accuracy basecalling was enabled through a graphics card running Guppy 5.0.16 (GeForce RTX 2070 Super; PopOS 20.04 LTS). What’s in my pot (Fastq WIMP 2021.11.16) analysis was done with EPI2ME, setting the threshold qscore at 10, and the minimum read length at 1,000. Furthermore, the obtained results were filtered to include only reads with WIMP score of at least 2,000 (this further removed ~10% of the remaining reads). The What’s in my pot version 2021.11.16 that we used for analysis compares the FASTQ sequences directly to the reference database, which is primarily microbial (bacteria, fungi, viruses), therefore, the host plant sequences do not align to it.

## 3 Results

Sample after 1 year of fermentation was not included in the metagenomics analysis as the DNA concentration was below required (<400 ng) even after clean-up procedure. The complete list of species identified per sample is presented in Supplementary Table.

**Figure 1:**
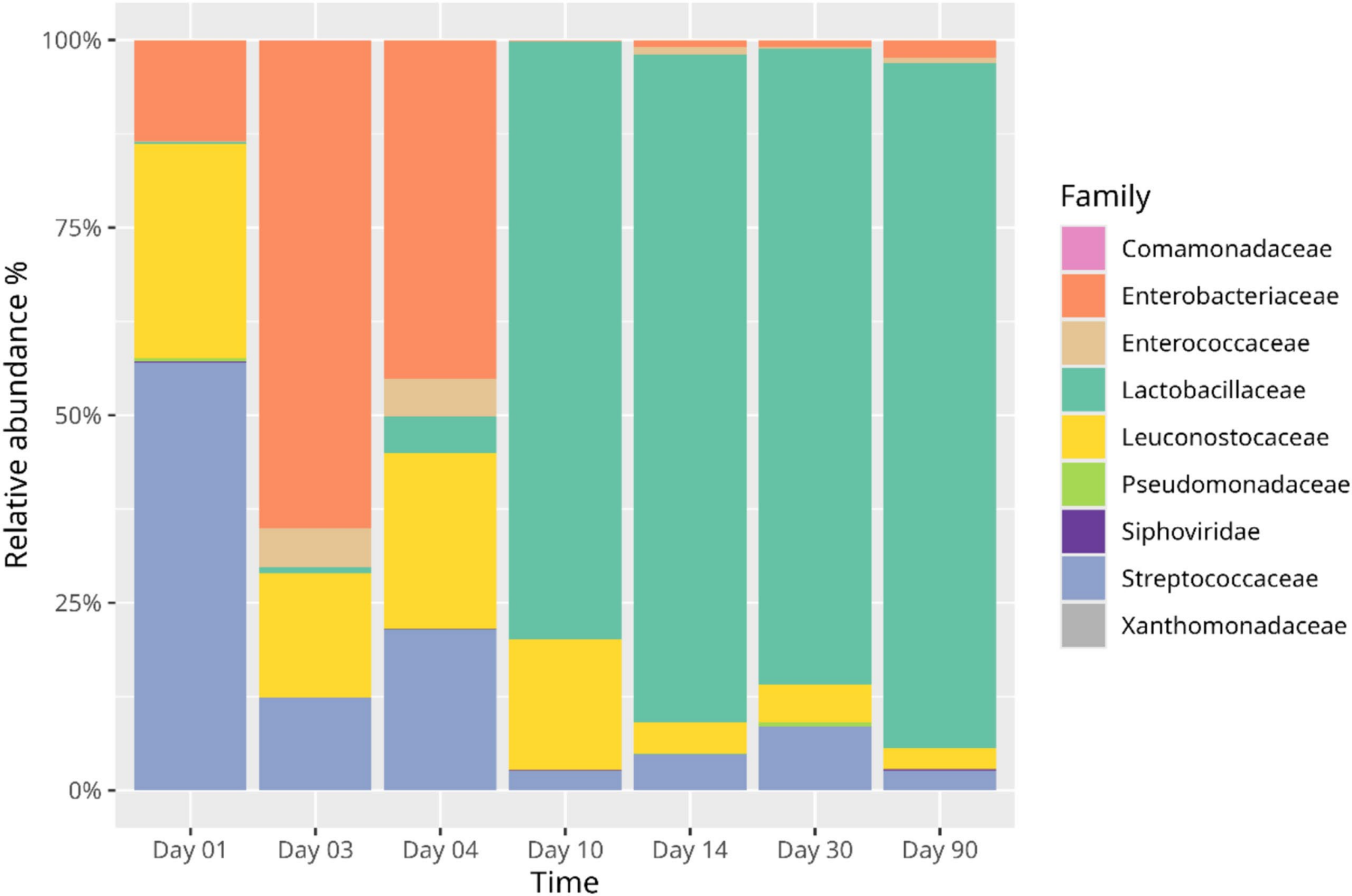
Changes in microbial composition in natural cucumber fermentation from own-cultivation. Unclassified species are not listed.

**Figure 2:**
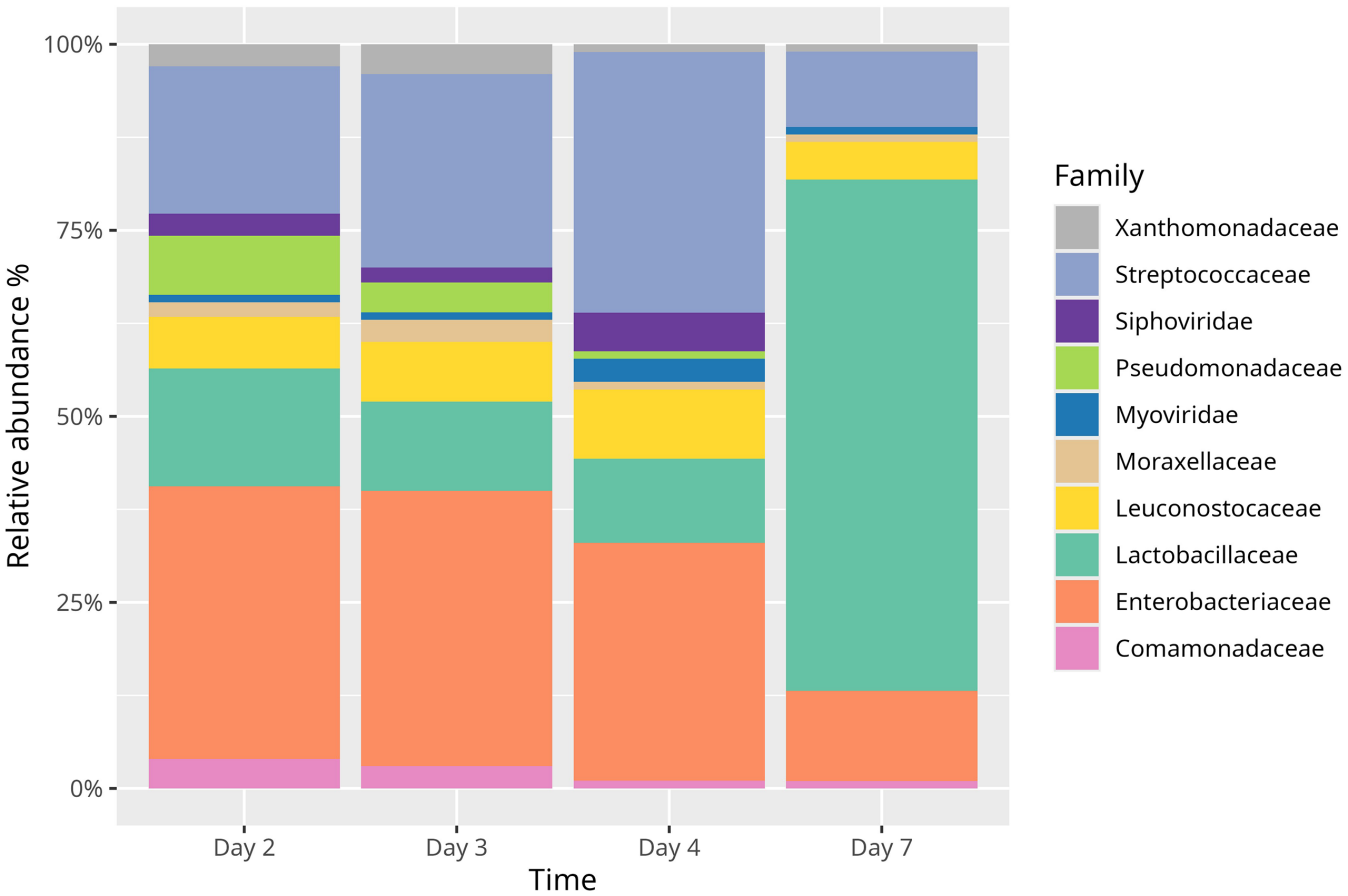
Changes in microbial composition in natural fermentation of commercial cucumber. Unclassified species are not listed.

The pattern of changes in microbial composition was different depending on the source of cucumbers (Figures 1, 2). The own-cultivation cucumber fermentation started mainly with *Lactococcus lactis* (47%) and *Leuconostoc mesenteroides* (22%), and to some extent *Rahnella aquatilis* (8%). On the third day, composition reversed, with an increase in *Rahnella aquatilis* (23%) and a gradual decline in *Leuconostoc mesenteroides* (11%) and *Lactococcus lactis* (8%). The leading player in fermentation on the third day was *Klebsiella* (42%), of which the *Klebsiella grimontii* was the most prevalent species (22%). On the fourth day, the genus *Rahnella* became more prevalent again (37%), as did *Lactococcus lactis* (24%). *Klebsiella* diminished (25%), and *Leuconostoc mesenteroides* were more or less stable (13%). On the tenth day, the main genera were *Lactiplantibacillus plantarum* (67%) and *Leuconostoc mesenteroides* (15%). On the fourteenth day, the leading role in fermentation belonged to *Companilactobacillus heilongjiangensis* (76%). On the thirtieth day of fermentation, the *Pediococcus* genus emerged (41%), with the main species being *Pediococcus pentosaceus* (23%), *Pediococcus damnosus* (10%), and *Pediococcus inopinatus* (8%). Genus *Pediococcus* remained the main genus until the ninetieth day (63%).

The sequencing of commercial cucumber fermentation brine identified *Myoviridae* and *Siphoviridae* with the highest abundance on the fourth day of fermentation. The commercial cucumber fermentation started with *Kluyvera intermedia* (12%) and *Lactococcus lactis* (10%). Six phages against the *Lactococcus* genus were present on that day at an abundance 2.0% (phiL47, 949, LW31, phiQ1, P596, P087). The sequencing identified eight other species, including phages against *Erwinia*, *Salmonella*, *Pectobacterium*, *Klebsiella*, *Escherichia coli*, *Edwardsiella*, *Enterobacter*, and *Cronobacter*. On the third day, the abundance of phages infecting *Lactococcus* decreased to 1.20%, and the abundance of the *Lactococcus* genus increased to 21.65%. On the fourth day, the *Lactococcus* genus reached over 30%, despite phages against *Lactococcus* being at their highest abundance 4.30% (phiL47, 949, LW31, WRP3, phiQ1, P087, AM6, 63301).

After a week of fermentation, the main genera were *Pediococcus pentosaceus* (30%) and *Lactiplantibacillus plantarum* (17%). The *Lactococcus* genus decreased to 9.05%, and the phages infecting *Lactococcus* decreased to 0.30%.

In the case of *Salmonella*, the bacteria were present from day 2 of fermentation (0.23%), along with phages infecting the *Salmonella* genus (0.23%). The highest abundance of bacteria was on the third day (0.36%), with a subsequent increase of specific phages (0.62%). The increase in phages against *Salmonella* continued through the fourth day of fermentation (0.87%) and putatively led to a decrease in bacteria on that day (0.20%). After 1 week of fermentation, *Salmonella* diminished to (0.08%), as well as the phages infecting the *Salmonella* genus, which dropped to almost initial values 0.29%.

The pattern seems similar for the *Erwinia* genus: the highest bacterial abundance was also on the third day (1.06%). The number of phages against *Erwinia* increased from the beginning (0.40%) to a peak on the fourth day (2.77%), while the number of bacteria decreased to 0.51%. After a week of fermentation, the *Erwinia* genus decreased to 0.46%, as well as phages infecting the *Erwinia* genus 1.11%.

In the own-cultivation cucumber brine, viruses were detected at a much lower frequency. Fungi were at undetected levels.

## 4 Discussion

Our study provides a metagenomic perspective on cucumber fermentation, addressing existing knowledge gaps in this field of research. We found that the occurrence of phages is more likely to happen in large-scale cucumber fermentations than in those of organic origin. In the commercial cucumber fermentation we found phages of 12 distinct genera (Supplementary Table). The top three most prevalent were against *Lactococcus* (average 1.95%), *Erwinia* (average 1.37%), *Salmonella* (average 0.50%). In the fermentation of own-cultivated cucumber the occurrence of phage was less than 1% (Supplementary Table). The phages were against *Lactococcus* (average 0.08%), *Erwinia* (average 0.04%) and *Salmonella* (<0.01%). It has previously been shown that 57 phages were isolated from brine over 90 days of commercial cucumber fermentation (Lu et al., 2012). Differences in farming practices likely explain this pattern, as *Enterobacteriaceae*—such as *Erwinia* and *Salmonella*—are more prevalent on conventionally grown fruits and vegetables than on those produced organically (Leff and Fierer, 2013). Factors such as type of fertilizer, pesticide use, soil type, water contamination introduce a significant variation to be accounted for before planning a study and drawing conclusions (Beuchat, 2006). Another source of differences in virome composition may be geographical location, as studies have shown that viruses are more strongly associated with the geographic origins of fermented products than bacteria [46].

Lu et al. (2020) suggested that the abundance of phages against *Enterobacteriaceae* and many other Gram-negative bacteria plays a crucial role in reducing those bacteria and promoting the dominance of LAB. The present report shows that even in the absence of phages (own-cultivation cucumbers <1%) the LAB dominance occurs, nevertheless. However, the amounts of *Enterobacteriaceae* on fermentation day 3 and 4 were higher in own-cultivation cucumbers (<1% phages detected) than commercial cucumbers (phages present from day 2). Therefore, the role of phages may be exaggerated, but it cannot be overlooked. Cardinali et al. (2024) pinpoint that the shift in *Enterobacteriaceae* is likely triggered by a rapid drop in pH caused by the LAB metabolic activity. Recent research has highlighted that exopolysaccharides (EPS) produced by LAB during fermentation exhibit antibacterial, antiviral and antifungal properties (Kavitake et al., 2023), which suggests that EPS may be involved in altering the balance of dominance from *Enterobacteriaceae* to LAB.

To our knowledge, this is the first report coagulase-negative cocci (namely, *Staphylococcus saprophyticus*, *Staphylococcus xylosus* and *Staphylococcus succinus*) in fermented cucumbers as in the case with other fermented foods. We detected *Staphylococcus saprophyticus* (0.01%) and *Staphylococcus schleiferi* (0.03%), both of which were only present in the brine of commercial cucumbers. Of these two, only *Staphylococcus saprophyticus* has a documented beneficial role in fermented food production. *Staphylococcus schleiferi* has been recently reported as pathogenic in milk and meat production (Supriya et al., 2024; Gundog et al., 2024). In our preliminary study, *Staphylococcus schleiferi* occurs only on the third day of fermentation, which might suggest that it is naturally eliminated in the process of fermentation. Additional research should investigate the mechanisms by which these microorganisms influence cucumber fermentation.

Many of the available data show commercial cucumber fermentations that employ cover brine containing preservatives (such as acid, salt, or vinegar) and well-established starter cultures for the production of safe foods. The present study demonstrates a natural process that lasts up to 90 days. Another cucumber fermentation study showed a gradual increase of LAB concentration from day one, peaking on day 7, and decreasing markedly on day 30 (Lu et al., 2020). In contrast, we did not observe any decrease in LAB. Instead, we show stable LAB concentration even in the 90th day of own-cultivation cucumber fermentation. Lu et al. (2020) observed no Gram-negative bacteria on day 7 and thereafter. Our study shows a similar drop in own-cultivation cucumber fermentation and a marked decrease of *Enterobacteriaceae* in commercial cucumber fermentation on day 7.

*Lactococcus lactis* has been found in the cover brine of fermented cucumbers produced at a commercial scale, although only during the first week of fermentation (Cardinali et al., 2024; Pérez-Díaz et al., 2017). Cardinali et al. suggested that this species might not survive as cucumber fermentation progresses. Indeed, in our study, the abundance of *Lactococcus lactis* decreases over time; however, it remains present even on the 30th and 90th days of fermentation (4.60 and 1.23%, respectively). We were unable to isolate enough DNA from the sample after 1 year of fermentation, indicating a substantial decline in microbial communities following prolonged fermentation.

Discrepancies in the literature focus on the role of *Leuconostoc mesenteroides* in cucumber fermentations. In low-temperature fermented cucumber with 2.5% salt and 15% sugar, *Leuconostoc mesenteroides* was identified as the dominant LAB, with an abundance of over 80% at the 8th day of fermentation (Kao et al., 2023). In contrast, our study showed *Leuconostoc mesenteroides* had an abundance of 15.41% (10th day) in own-cultivation cucumber brine and 0.12% (7th day) in commercial cucumber brine. Decreased values of *Leuconostoc mesenteroides* as the cucumber fermentation proceeded were also reported earlier, even in higher salinity (Singh and Ramesh, 2008; Egbe et al., 2017). This study contributes to the body of knowledge by providing an additional perspective.

Thanks to the metagenomic approach, we have been able to look beyond the bacterial level. Specifically, we detected phages whose abundance and diversity vary depending on the cucumbers’ origin (organic vs. large-scale farming). This finding, along with the maturity of fermentation products, has practical implications for designing future studies and for direct consumers. Our data also demonstrate that even in the near absence of phages (as observed in home-grown cucumbers, <1%), LAB dominance occurs. This perspective adds to the ongoing discussion about the role of phages in maintaining the balance between *Enterobacteriaceae* and LAB during plant fermentation.

## Data Availability

The datasets presented in this study can be found in online repositories. The names of the repository/repositories and accession number(s) can be found in the article/Supplementary material.
